# Florid ascites associated with alcohol use disorder without cirrhosis

**DOI:** 10.1093/qjmed/hcaf069

**Published:** 2025-03-14

**Authors:** Ami Schattner, Ina Dubin, Livnat Uliel

**Affiliations:** Department of Medicine, Laniado Hospital, Netanya, Israel; Adelson School of Medicine, Ariel University, Ariel, Israel; Faculty of Medicine, Hebrew University and Hadassah Medical School, Jerusalem, Israel; Department of Medicine, Laniado Hospital, Netanya, Israel; Adelson School of Medicine, Ariel University, Ariel, Israel; Adelson School of Medicine, Ariel University, Ariel, Israel; Department of Imaging, Laniado Hospital, Netanya, Israel

Learning points for clinicians

• Alcohol-associated liver disease represents a spectrum of liver injury ranging from asymptomatic steatosis, through alcoholic hepatitis (AH) to overt cirrhosis and liver failure.
• Portal hypertension without cirrhosis is mostly associated with pre-sinusoidal or post-sinusoidal vascular obstruction.
• Portal hypertension with associated marked ascites can rarely develop in the setting of isolated acute AH.

**Figure 1.**
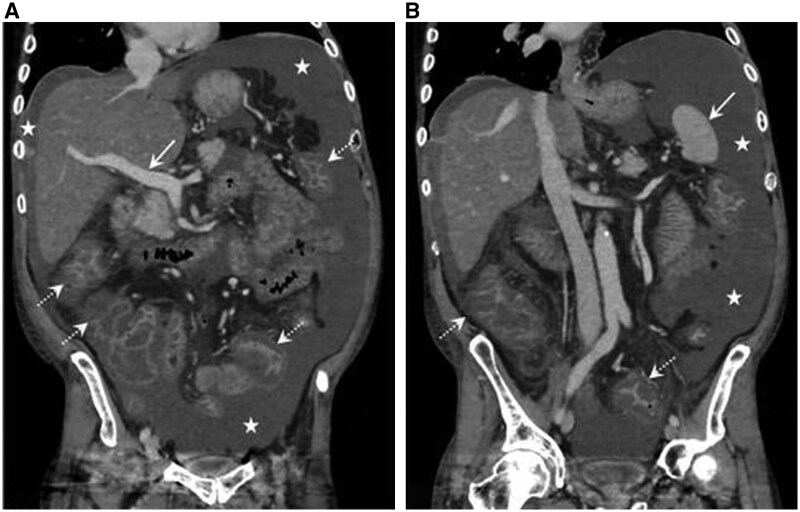
Coronal post-contrast computed tomography images at the time of admission reveal a large amount of ascites (asterisks). Recanalization of the umbilical vein (a collateral, secondary to portal hypertension) was clearly demonstrated (not shown). The liver is normal in size and contour, showing diffuse fat infiltration. The main portal vein is well visualized (arrow in A), with no evidence of portal or hepatic veins thrombosis. No splenomegaly is noted (arrow in B). Acute pancolitis is evident, as demonstrated by diffuse wall thickening of the large bowel loops (dashed arrows).

A 51-year-old homeless, street-dwelling man with a long history of alcohol abuse (0.5–1 bottle vodka/day) was admitted, complaining of increasing diffuse abdominal pain and girth over 2 weeks, and acute diarrhoea, vomiting and weakness. Vital signs were normal (body mass index 21.4). Liver tenderness, ascites with caput-medusae and ankle oedema were found. Clubbing, palmar-erythema, spider-angiomas, gynaecomastia or testicular atrophy was not seen. Chest X-ray was normal. Abdominal Doppler ultrasound and computed tomography demonstrated ascites with open/normal-flow portal and hepatic veins, and normal-appearing liver (with steatosis) and spleen. Severe diffuse enterocolitis was also seen (Figure 1).

Hb was 10.6 g/dl (mean corpuscular volume 118 fl), white blood cells 7.1 × 10^9^/l, platelets 87 × 10^9^/l and alcohol level 178 mg/dl. Blood gases, glucose and prothrombin time (12.8 seconds/85%, then 107%) were normal. Hypokalaemia (2.6 mEq/l), hypomagnesaemia (1.25 mg/dl) and hyponatraemia (128 mEq/l) were associated with hypoalbuminaemia (2.1 g/dl), C-reactive protein (CRP) 27.1 mg/l (*N* < 5) and stool molecular-panel detected enteropathogenic *Escherichia coli*. In an emergency department visit *1 month prior* after a fall, CRP was 0.6 mg/l, serum albumin was 3.6 g/dl and gamma-glutamyltransferase (GGT) 258 IU/l (*N* < 30) was the only abnormality. Currently, GGT was 477 IU/l, alkaline-phosphatase 212 IU/l (*N* < 120), bilirubin 5.3 mg/dl (*N* < 1.2), aspartate-aminotransferase 148 IU/l and alanine-aminotransferase 43.4 IU/l (ratio 3.4), consistent with acute alcoholic hepatitis (AH). Pancreatic enzymes were normal as was urinalysis, screening for hepatitis viruses, drug screen, autoantibodies and serum-B12. Folate was 1.58 ng/ml (*N* > 4.6). Ceftazidime and metronidazole were given, fluid/electrolyte deficiencies were corrected, and thiamine was supplemented. Diarrhoea resolved, electrolytes normalized, albumin increased (3.4 g/dl) and liver enzymes decreased, with CRP 3.4 mg/dl.

Ascitic fluid (9 l, slowly removed) yielded albumin 0.2 g/dl and serum-ascites-albumin-gradient (SAAG) 2.2, lactate dehydrogenase 41 IU/l, with 43 mononuclear cells/µl and negative microbiology. Endoscopy revealed gastritis, but no varices. Echocardiography was normal. Low-salt diet and furosemide/spironolactone were given. The patient gave signed consent for this publication but adamantly refused liver biopsy, and an alcohol use disorder-treatment centre referral. He was discharged to a long-term care facility on the 16th hospital day with diminishing ascites on diet and furosemide/spironolactone. Transient elastography (FibroScan) 1 month later yielded 8.5 kPa, indicating fibrosis stage F3.

Our patient’s acute bacterial pancolitis was associated with pronounced electrolyte abnormalities and hypoalbuminaemia, which resolved with treatment. The transudative (SAAG 2.2) massive ascites did not.

Contrary to expectations, considering his heavy alcohol abuse,^1^ he had steatosis but no evidence of liver cirrhosis. Portal hypertension seemed quite new, absent in his recent emergency department visit. Cirrhosis stigmata were absent; the biosynthetic functions of the liver were preserved (near-normal prothrombin time and serum albumin after recovery). Neither splenomegaly nor oesophageal varices were found; and the blood count showed only transient thrombocytopenia and macrocytic anaemia, due to bone-marrow effects of alcohol/folate deficiency. Finally, the liver appearance on imaging was also not compatible with cirrhosis,^2^ and FibroScan was below the 12.5 kPa cut-off for F4/cirrhosis. However, ascites due to portal hypertension remained a major finding.

Non-cirrhotic portal hypertension is well-described, and mostly associated with vascular obstruction which can be either pre-sinusoidal (e.g. portal vein thrombosis) or post-sinusoidal (e.g. hepatic vein thrombosis, veno-occlusive disease).^3^^,^^4^ Neither is associated with alcohol abuse without cirrhosis, and both were ruled out by the imaging studies, leaving intra-hepatic sinusoidal portal hypertension as the cause. Diffuse, alcohol-associated steatosis preceded his AH and portal hypertension, both absent 1 month prior. The observation that AH could induce sinusoidal damage sufficient to increase portal pressure and cause ascites is intriguing, more so since it was not clinically severe (low Maddrey’s discriminant function).^1^ The transient, infection-associated hypoalbuminaemia, likely contributed to ascites formation but cannot have caused portal hypertension.^5^ Diverse patterns of histological sinusoidal pathology in AH have been reported, including fibrosis, defenestration and compression by enlarged hepatocytes.^3^ Our report attests to the rapidity in which alcoholic liver disease can progress, stressing the paramount value of abstinence.
